# A new reassortment of influenza A (H7N9) virus causing human infection in Beijing, 2014

**DOI:** 10.1038/srep26624

**Published:** 2016-05-27

**Authors:** Yuhai Bi, Jingyuan Liu, Haofeng Xiong, Yue Zhang, Di Liu, Yingxia Liu, George F. Gao, Beibei Wang

**Affiliations:** 1 Shenzhen Key Laboratory of Pathogen and Immunity, Shenzhen Third People’s Hospital, Shenzhen 518112, China; 2 CAS Key Laboratory of Pathogenic Microbiology and Immunology (CASPMI), Institute of Microbiology, Chinese Academy of Sciences, Beijing 100101, China; 3 Center for Influenza Research and Early-warning (CASCIRE), Chinese Academy of Sciences, Beijing 100101, China; 4 Intensive Care Unit, Beijing Ditan Hospital, Capital Medical University, Beijing 100015, China; 5 Institute of Infectious Diseases, Beijing Ditan Hospital, Capital Medical University, Beijing 100015, China; 6 Beijing Key Laboratory of Emerging Infectious Diseases, Beijing 100015, China; 7 Network Information Center, Institute of Microbiology, Chinese Academy of Sciences, Beijing 100101, China

## Abstract

A 73-year-old man was confirmed to have an influenza A (H7N9) virus infection, and the causative agent A/Beijing/02/2014(H7N9) virus was isolated. Genetic and phylogenetic analyses revealed that the virus belonged to a novel genotype, which probably emerged and further reassorted with other H9 or H7 viruses in poultry before transmitting to humans. This virus caused a severe infection with high levels of cytokines and neutralizing antibodies. Eventually, the patient was cured after serially combined treatments. Taken together, our findings indicated that this novel genotype of the human H7N9 virus did not evolve directly from the first Beijing isolate A/Beijing/01/2013(H7N9), suggesting that the H7N9 virus has not obtained the ability for human-to-human transmissibility and the virus only evolves in poultry and then infects human by direct contact. Hence, the major measures to prevent human H7N9 virus infection are still to control and standardize the live poultry trade. Early antiviral treatment with combination therapies, including mechanical ventilation, nutrition support and symptomatic treatment, are effective for H7N9 infection.

Since March 2013, novel influenza A (H7N9) viruses have emerged in China and spread quickly, causing severe respiratory disease in humans^1^,^2^. As of 20 January 2016, a total of 693 laboratory-confirmed cases had been reported, and there were 277 deaths (http://www.who.int/influenza/human_animal_interface/HAI_Risk_Assessment/en/). Recent studies showed that the internal genes of the H7N9 virus have continued to undergo dynamic reassortments with the poultry H9N2 viruses^3^,^4^,^5^,^6^. According to the evolutionary distance and reassortment style, the H7N9 viruses were classified into 27 genotypes within the first three months of the initial outbreak and into 48 more genotypes to date by our and another group respectively^3^,^6^. Among the genotypes, the G0 or W1 genotype (represented by A/Anhui/1/2013) acts as the dominant virus cluster in humans^3^,^6^. None of the G4, G5 and G6 viruses, which have 4, 5 and 6 phylogenetically different internal genes from G0, has been observed in humans based on surveillance data from 109 isolates^3^. The genotypic diversity would possibly possess varied virulence and host adaptations in humans because extensive surveillance on patients with flu-like symptoms revealed H7N9 infections with only mild to moderate symptoms^7^. On 12 April 2013, the first human H7N9-infection case in Beijing with the A/Beijing/01-A/2013(H7N9) virus (abbreviated as BJ01 thereafter) was identified^8^,^9^. On 5 February 2014, a new H7N9-infection case was confirmed in Beijing. The gene evolution of the H7N9 viruses in Beijing needs to be investigated for further prevention and control of the H7N9 infection.

## Results

### Case description and treatment

A 73-year-old man who worked as a live poultry seller and butcher in Beijing, China was diagnosed with an influenza A (H7N9) virus infection confirmed by detection of the H7N9 virus in the laboratory. The patient was an alcoholic with a past medical history of chronic bronchitis and coronary heart disease. The illness began with flu-like symptoms, including a high fever (38.3 °C), cough with yellow-white phlegm and feeling fatigued on 30 January 2014. Because the detection of the influenza A virus universal antigen was negative on the throat-swab by means of the immune colloidal gold technique and the radiologic findings revealed bronchitis, the patient was treated with anti-infective therapy by an intravenous injection of moxifloxacin. However, that treatment did not take effect, and the symptoms gradually worsened.

On 5 February 2014, the patient appeared with hyperpyrexia (maximum temperature 40.0 °C), coughing with bloody sputum and dyspnoea with a low oxygen saturation (88.8%). The H7N9 viral RNA was positive in the oropharynx swab confirmed by the real-time RT-PCR method according to the protocol of the Chinese CDC^10^. The patient was transferred into the intensive care unit (ICU) of Beijing Ditan Hospital, Capital Medical University. The case was diagnosed as a laboratory-confirmed case of influenza A (H7N9) infection with severe pneumonia combined with the complications of acute respiratory failure, septic shock, stress ulcer and acute renal failure. Antiviral treatment (oseltamivir) with combination of antibiotics (Sulperazon), a gastric acid secretion inhibitor (omeprazole), mechanical ventilation, continuous renal replacement, supportive nutrition therapy and symptomatic treatment were given. On 12 February 2014, the H7N9 viral nucleic acid was negative when detecting the tracheal aspirate specimens by real-time RT-PCR. On 12 March 2014, the infection symptoms and the respiratory function improved, and the circulation situation tended to be stable. After approximately four months of treatment, the patient recovered and was discharged from hospital on 6 June 2014 (Table 1).

### Virology analyses

A H7N9 virus, A/Beijing/02/2014(H7N9) (abbreviated as BJ02 thereafter), was isolated and identified after one passage propagation in eggs. To further study the gene evolution of the H7N9 virus, the whole genome was amplified and sequenced. Genetic alignments showed that the HA, NA, PB1, PA, NP and NS genes possessed the highest genetic similarities (99.33–99.74%) with other H7N9 virus genes. However, the PB2 and M genes possessed the highest nucleotide similarities (99.39% and 98.73%, respectively) with H9N2 isolates (Table 2). Furthermore, the genetic homology between BJ02 and BJ01 displayed some diversity because there are 96.27% and 97.66% nucleotide similarities, respectively, of the PB2 and M genes (Table 2). These nucleotide identities suggested that BJ02 might not be evolved directly from BJ01.

**Figure 1:**
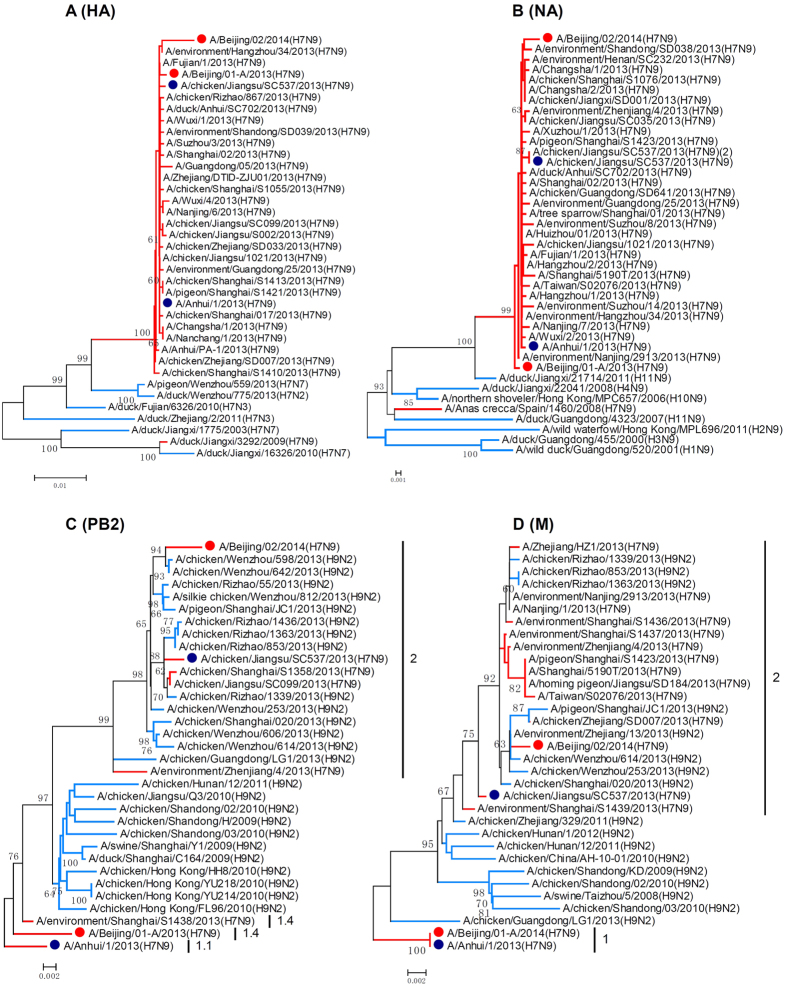
Phylogenetic analysis of HA, NA, PB2 and M genes of H7N9 influenza viruses. The phylogenies were inferred by the Maximum Composite Likelihood model of the Neighbor-Joining algorithm with the software MEGA5, and 1000 bootstrap replicates were applied. The branches for the H7N9 viruses are in red; H7Nx (**A**), HxN9 (**B**) and H9N2 (**C**,**D**) viruses are in blue; the red dots represent the Beijing isolates. The blue dots represent the representative isolates, A/Anhui/1/2013(H7N9) and A/chicken/Jiangsu/SC537/2013(H7N9).

**Figure 2:**
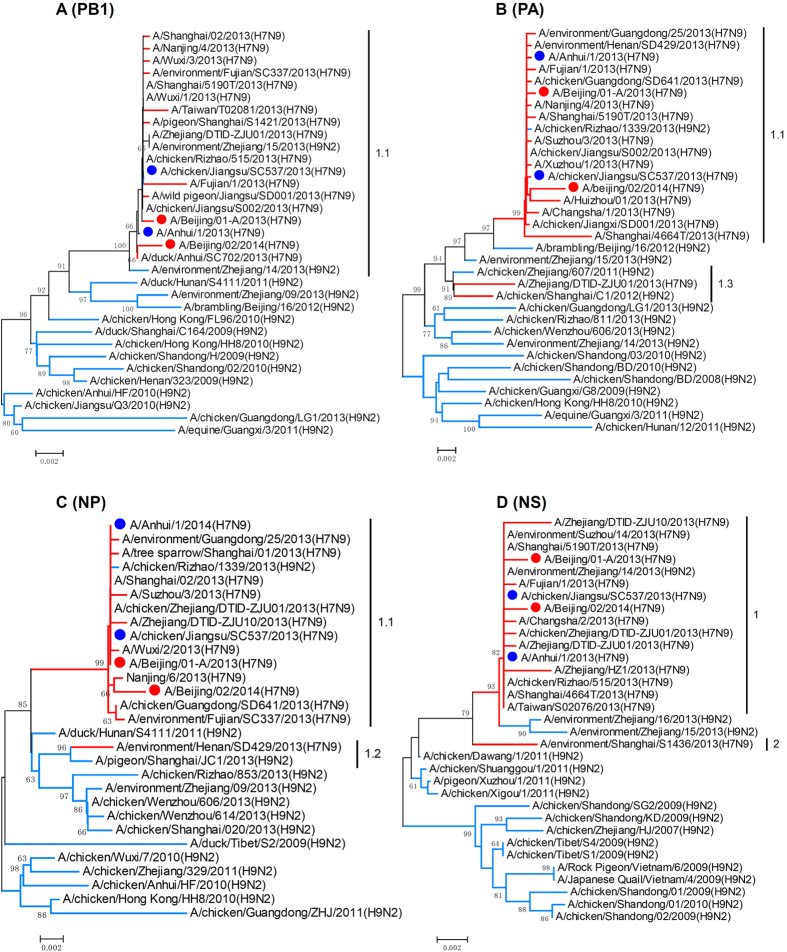
Phylogenetic analysis of PB1, PA, NP and NS genes of H7N9 influenza viruses. The phylogenies were inferred by the Maximum Composite Likelihood model of the Neighbor-Joining algorithm with the software MEGA5, and 1000 bootstrap replicates were applied. The branches for the H7N9 and H9N2 viruses are in red and blue, respectively. The red dots represent the Beijing isolates. The blue dots represent the representative isolates, A/Anhui/1/2013(H7N9) and A/chicken/Jiangsu/SC537/2013(H7N9).

The phylogenetic analyses showed that the HA and NA gene sequences of BJ02 clustered with previously identified human-infecting H7N9 viruses (Fig. 1A,B). The phylogenies of the PB2 and M genes documented that BJ02 and BJ01 fell into different clades, and this finding implied that different evolutionary pathways might behind these two viruses (Fig. 1C,D). According to the genotypic assignment in the previous study^3^, the PB2, PB1, PA, NP, M and NS genes of BJ02 were assigned to clades 2, 1.1, 1.1, 1.1, 2 and 1, respectively, in the phylogenetic trees, and these results suggest that BJ02 should be designated as Genotype 2.9 (G2.9). BJ01 was designated as Genotype 1.5 (G1.5) because its internal genes grouped into clades 1.4, 1.1, 1.1, 1.1, 1 and 1 (Figs 1C,D and 2). This case was the first human infection caused by the G2.9 virus. Notably, the G2.9 virus was previously isolated from poultry in the Jiangsu province in 2013 (A/chicken/Jiangsu/SC537/2013(H7N9), SC537). BJ02 and SC537 shared high genetic similarities (Table 2). However, the PB2 and M genes of BJ02 clustered together with other avian-source H9N2 viruses (Fig. 1C,D). All of these findings suggested that this novel genotype virus should emerge and evolve in poultry prior to transmission to humans.

### Neutralizing antibody and cytokine evaluation

**Figure 3:**
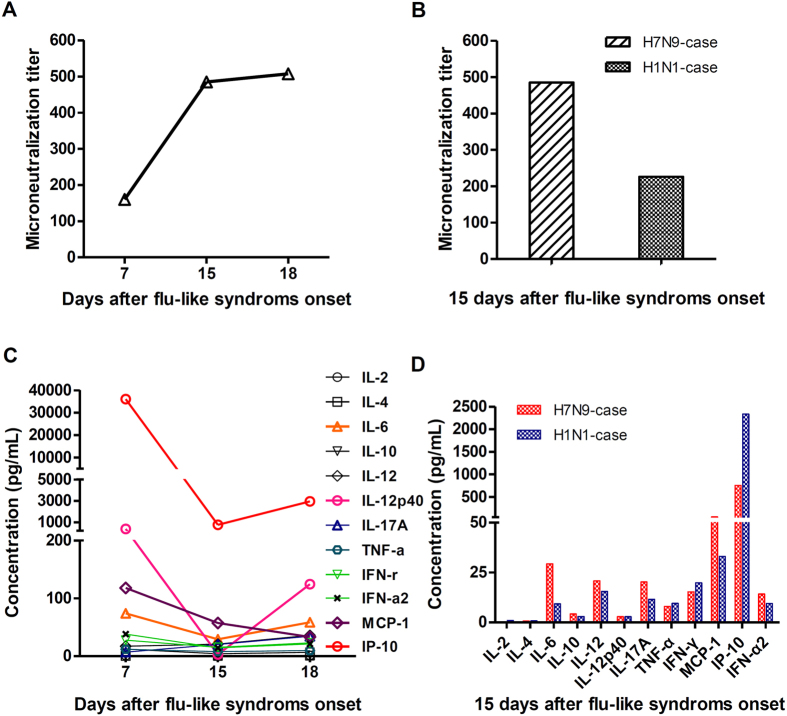
The kinetic changes of neutralization antibodies and cytokines in the patient induced by A/Beijing/02/2014(H7N9). The sera of the H7N9 patient were collected on 7, 15 and 18 d.a.o. and were used to detect the changing MN titres (**A**) and cytokines (**C**). The serum of a pandemic-H1N1 patient was collected on 15 d.a.o. as a control and was compared to the results of the H7N9 patient (**B,D**).

The level of serum neutralizing antibody (NAb) and cytokines in flu patients play pivotal roles in disease progression and recovery^10^,^11^,^12^,^13^,^14^,^15^,^16^,^17^,^18^,^19^. For this reason, the kinetic changes of NAb microneutralization (MN) titre and cytokines in the patient’s serum were detected. As shown in Fig. 3A, the seroconversion (>1:40) of the MN titre in this case appeared on day 7 after the onset of flu-like symptoms. Then, the MN titre raised to a plateau with a significantly high level (1:480~1:510) during 15~18 d.a.o. (days after disease onset, d.a.o.). The MN titre of the H7N9 patient was higher than that of the contemporaneous H1N1 patient on 15 d.a.o. (Fig. 3B).

The evaluation of serum cytokines in the H7N9 patient revealed that the levels of IL-6, IL-12p40, MCP-1 and IP-10 were significantly increased on 7, 15 and 18 d.a.o. compared with the normal ranges, whereas the IFN-γ, IFN-α and IL-17A levels did not notably increase (Fig. 3C). The levels of IL-6, IL-12p40, and IP-10 displayed an increase-decrease-increase tendency, and the highest levels were on 7 d.a.o., then decreased to the lowest points on 15 d.a.o., and subsequently showed increasing levels on 18 d.a.o. Additionally, most of the detected cytokines in the H7N9 case showed higher levels than in the contemporaneous severe H1N1 case on 15 d.a.o. (Fig. 3D), which suggested that the H7N9 virus might cause higher cytokine secretions than the H1N1 virus, although the case number in our study was limited.

## Discussion

The present data documented that the novel genotype G2.9 of the H7N9 virus (BJ02) could infect and cause severe disease in humans. In addition to the G1.5 virus (BJ01), there are at least two genotypes of H7N9 virus existing in Beijing. To date, only one G1.5 and one G2.9 virus from humans have been isolated; and the other G2.9 strain (SC537) was first isolated from chickens. Although high homology is shared between BJ02 and SC537 (Table 2), the PB2 and M genes of the BJ02 virus clustered with other avian-source H9N2 or H7N9 viruses (Fig. 1C,D). These data indicate that BJ02 likely evolved from a novel genotype found in chickens (SC537) that then reassorted with other H9 or H7 virus in birds before being transmitted to humans. This finding suggested that the reassortment with other subtype viruses in avians is still the major evolutionary path of the H7N9 virus; then, sensitive people would be infected after exposure to the mutated viruses. Hence, diverse H7N9 genotypes or reassortants were consecutively isolated in humans. To standardize or close the live poultry trade would be the most efficient way to prevent and control the human-avian influenza virus infection disease.

To date, a total of 29 genotypes of H7N9 viruses, including BJ01 (G1.5) and BJ02 (G2.9), have been identified according to our classification and nomenclature system^3^. However, there are only 11 genotype viruses isolated and identified in humans^3^. This finding suggested that diverse genotype viruses should possess heterogenic host-specific preference and pathogenicity for humans. The BJ01 virus only caused the patient to have a mild to moderate respiratory syndrome^2^, whereas the BJ02 virus tended to cause a severe disease. The underlying mechanisms need to be further clarified.

In addition, the oseltamivir resistance mutation (R292K) in the NA protein of BJ02 could not be found after 3 days of oseltamivir usage; however, the mutation emerged in the A/Shanghai/1/2013(H7N9) strain just after one day treatment with oseltamivir^10^,^20^. This finding further suggested that diverse biologic characteristics might appear in different H7N9 viruses.

In general, viral and host factors contribute to disease severity and outcomes. A previous multivariate analysis revealed that the presence of coexisting medical conditions (such as chronic heart disease and chronic obstructive pulmonary disease) were the only independent risk factors for severe illness with H7N9 infection^1^. Recent studies found that the presence of host genetic factors might be closely related to H7N9 influenza disease susceptibility and/or severity^21^,^22^. The IFN-induced transmembrane protein-3 (IFITM3) C/C genotype was reported to be associated with severe clinical outcomes, as reflected by a higher viral load, more rapid progression to ARDS, higher cytokine/chemokine levels, and an increased mortality rate after H7N9 infection^22^. Recently, increasing data showed that elevated concentrations of inflammatory cytokines/chemokines (especially IL-6, IL-8, MIP-1β, IP-10, MIF, SCF, MCP-1, and HGF) in the infected lung and plasma (hypercytokinaemia) are highly positively linked to disease severity in H7N9 infected patients^23^,^24^,^25^,^26^. Cytokine production is closely related to the severity of host lesions due to influenza virus infection^27^,^28^,^29^. Proper cytokine expression is necessary for disease recovery, defending against virus infection, and recruiting inflammatory cells to the sites of infection through a delicate balance between pro- and anti-inflammatory mediators^30^. However, cytokine over-expression breaks the immunologic balance, which causes systemic inflammation, acute organ dysfunction and even death^11^,^31^,^32^. H7N9-infected patients present the manifestations of acute respiratory disease syndrome (ARDS) and hypercytokinaemia^10^,^12^, which also were observed in the present case. In the present study, the H7N9 patient displayed the highest levels of IP-10, IL-6, IL-12p40 and MCP-1 secretion in the serum obtained during the acute phase of the disease (7 d.a.o.). Notably, the elevation of the chemokine IP-10 was the most robust among all of the detected cytokines and chemokines. Because IP-10 is a critical player in the induction of lung injury, its upregulation in H7N9 infection might be positively linked to disease severity, and this molecule could be a sensitive outcome predictor^24^. Although the case number was limited in our study to compare the serum cytokine levels between H7N9 and H1N1 infected patients, a similar profile of mediator secretion patterns in H7N9 and H1N1 infected patients has been seen by other groups with more influenza A patients^1^,^24^. Hence, the higher level of cytokine secretion might explain the more severe syndromes caused by H7N9 virus infection than by the contemporaneous H1N1 virus. It should be mentioned that there are also some different changes between our study and other studies in the cytokines for the H7N9 infections^12^,^33^,^34^,^35^,^36^. This finding might be related to the individual differences of host immune responses and the virus pathogenic characteristics, which should be further determined with more sample cases in the future.

Although this was a severe H7N9-infected case with cytokine storm-like appearances and multiple organ failure, the patient was eventually cured after combination therapy with antivirals, mechanical ventilation, supportive nutrition and symptomatic treatment. In our study, the 73-year-old patient was not prescribed oseltamivir until day 6 d.a.o. because the H7N9 virus was not identified at the onset of his illness. According to the clinical finding reports of the H7N9-infected patients in 2013, the median time of the initiated antiviral therapy was 7 days after the onset of illness^1^. Although it is difficult to identify, diagnose and initiate antiviral therapy within 3 d.a.o., neuraminidase inhibitors should be employed as soon as possible (ideally, within 48 hours following symptom onset) to maximise the therapeutic benefits and reduce the incidence of severe illness. To achieve this purpose, we need to further optimize the diagnostic tools for influenza-infection detection.

A previous study showed that the early and rapid induction of NAb was correlated significantly with better clinical outcomes^14^,^37^. As shown in our study, the seroconversion of NAb in this H7N9 case appeared on day 7 d.a.o., and the MN titres increased rapidly until reaching a plateau at 15~18 d.a.o. with a significantly high level (1:480~1:510). Hence, the quickly increased NAb level probably contributed to the virus clearance and the patient’s recovery.

The H7N9 influenza virus is spreading, evolving and becoming widespread among chickens in China^6^. Fortunately, the virus has not completely obtained the ability for human-like receptor binding and human-to-human transmission. Moreover, the H7N9 virus still mainly originates and evolves in avian species; therefore, it is possible to intercept the interspecies transmission by controlling or standardizing live poultry trade. Additionally, we should persistent in monitoring the gene evolution of the H7N9 virus isolated from humans and avian species and should optimise diagnostic tools, develop antiviral drugs and spread effective combination therapies for H7N9 infections.

## Materials and Methods

### Clinical samples and ethical approval

A suspected case of H7N9 influenza virus infection was confirmed by a real-time RT-PCR assay in the Beijing Centers for Disease Control and Prevention (CDC). The epidemiologic and clinical data were collected. A confirmed case was defined as evidence of pneumonia with H7N9 viral RNA or isolation of H7N9 virus from respiratory specimens. Informed consent was obtained from all participating individuals. This experimental protocol was approved by the local Ethics Committee of Beijing Ditan Hospital, Capital Medical University. The methods were carried out in accordance with the approved guidelines.

### Virology analyses

An oropharynx swab, collected on day 8 after the onset of flu-like symptoms, was used for virus isolation. The virus was propagated in 10-day-old specific pathogen free (SPF) embryonated chicken eggs and MDCK cells for 48 to 72 hours at 35 °C, respectively. For genetic analysis, the complete gene segments were amplified using improved primers (Table S1) based on previous reports^38^,^39^ and were sequenced by an ABI 3730XL automatic DNA analyser (Applied Biosystems, Foster City, CA, United States). Genetic identification and homology of the isolate were performed using the BLAST method in NCBI. A phylogenetic analysis was constructed using the maximum likelihood method with MEGA5 (http://www.megasoftware.net). The genotype analysis of the H7N9 virus was classified by the criteria previously described^3^.

### Neutralizing antibody and cytokine evaluation

Sera from the H7N9 patient were consecutively collected on 7, 15 and 18 d.a.o. for kinetic evaluations of the neutralizing antibodies and cytokine levels. A pandemic H1N1 severe case, onset on 2 February 2014, was used as a control, and the serum was collected on 15 d.a.o. The neutralizing antibodies (NAbs) were detected by Microneutralization Assays with A/Anhui/1/2013 (H7N9) and A/California/04/2009 (H1N1) viruses in MDCK cells, according to the previously described method^40^. The levels of cytokines in the H7N9 patient’s serum were detected by the Bio-Plex Human Cytokine Panel (Bio-Rad Laboratories, Inc.) according to the instructions.

## Additional Information

**How to cite this article**: Bi, Y. *et al*. A new reassortment of influenza A (H7N9) virus causing human infection in Beijing, 2014. *Sci. Rep*. **6**, 26624; doi: 10.1038/srep26624 (2016).

## Supplementary Material

Supplementary Information

## Figures and Tables

**Table 1:** Demographics and clinical information of the H7N9 virus-infected patient.

Sex	Age, (y)	Underlying medical disorders	Days from disease onset to										Clinical Outcome (days from disease onset)
			Admission	Virus confirmation	Fever	Cough	Hypoxemia	Pneumonia	ARDS	Initiation of Oseltamivir	Mechanical ventilation	Disappearance of virus	
Male	73	Chronic bronchitis, Coronary heart disease	6	6	0	0	6	6	6	6	6	13	discharged (127)

The symptoms started on 30 January 2014, and that day was set as the disease-onset day.

**Table 2:**
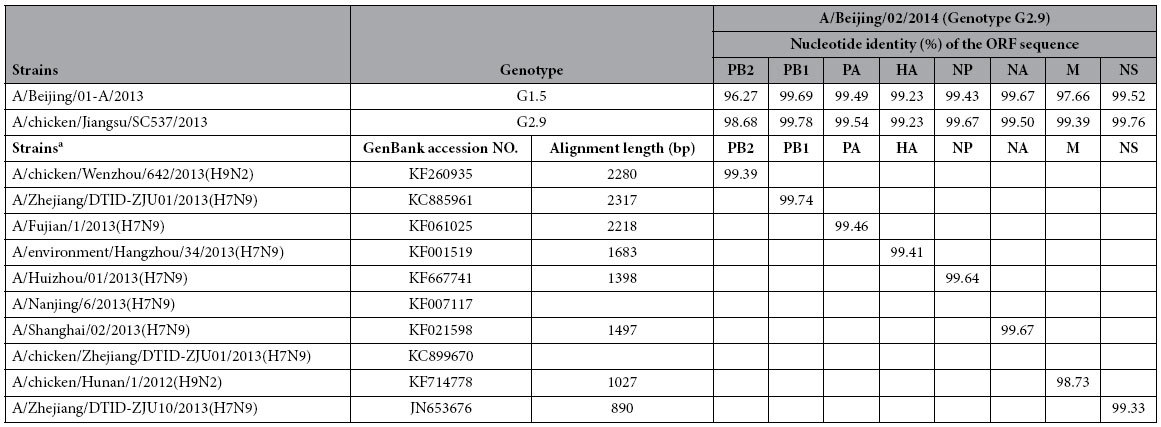
Genetic similarities between the A/Beijing/02/2014 virus and other H7N9 or H9N2 viruses.

^a^The alignment was executed on the web site http://blast.ncbi.nlm.nih.gov/Blast.cgi and performed on April 20, 2014.Each genetic ORF sequence of the A/Beijing/02/2014(H7N9) virus was used with Blast to find highly similar sequences, and the strain that possesses the highest genetic similarity in the NCBI database was shown.
