# Supplementary material for: A new reassortment of influenza A (H7N9) virus causing human infection in Beijing, 2014
Source: Sci Rep. 2016 May 27;6:26624. doi: 10.1038/srep26624 (PMC4882526; doi:10.1038/srep26624)
Supplement: Supplementary Information [file srep26624-s1.pdf]

A new reassortant of influenza A (H7N9) virus causing human infection in Beijing, 2014

Yuhai Bi[1,2,3], Jingyuan Liu[4], Haofeng Xiong[4], Yue Zhang[5,6], Di Liu[2,3,7], Yingxia Liu[1], George F. Gao[1,2,3] and Beibei Wang[5,6*]

Table S1. The primers used for amplifying the eight gene segments

Genes	Primers	Genes	Primers
**HA**	HA-F: 5'-gTC TCA ggg AgC AAA AgC Agg gg-3'<br>HA-R: 5'-Tgg AgT AgA AAC AAg ggT gTT TT-3'	**PA**	PA-S-F: 5'-ggg AgC gAA AgC Agg TAC-3'<br>PA-S-R: 5'-TNg TYC TRC AYT TgC TTA TCA T-3'<br>PA-X-F: 5'-CAT TgA ggg CAA gCT TTC-3'<br>PA-X-R: 5'-CCg gAg TAg AAA CAA ggT ACT T-3'
**NA**	NA-F: 5'-AT ggA gCA AAA gCA ggA gT-3'<br>NA-R: 5'-ggCC AgT AgA AAC AAg gAg TTT TTT-3'	**NP**	NP-F: 5'-TAgg A gCA AAA gCA ggg TA-3'<br>NP-R: 5'-Tgg AgT AgA AAC AAg ggT ATT TTT-3'
**PB2**	PB2-S-F: 5'-TAgg AgC gAA AgC Agg TC-3'<br>PB2-S-R:5'-TCY TCY TgT gAR AAY ACC AT-3'<br>PB2-X-F: 5'-TAY gAR gAR TTC ACA ATg gT-3'<br>PB2-X-R: 5'-ggA gTA gAA ACA Agg TCg TTT-3'	**M**	M-F:5'-ACgg A gCA AAA gCA ggT Ag-3'<br>M-R: 5'-Cgg AgT AgA AAC AAg gTA gTT TTT-3'
**PB1**	PB1-S-F: 5'-ATT TAg CgA AAg CAg gCA-3'<br>PB1-S-R:5'-TTR AAC ATg CCC ATC ATC AT-3'<br>PB1-X-F: 5'-ARA TAC CNg CAg ARA TgC T-3'<br>PB1-X-R: 5'-Tgg AgT AgA AAC AAg gCA TTT-3'	**NS**	NS-F: 5'-TggA A gCA AAA gCA ggg Tg-3'<br>NS-R: 5'-Tgg AgT AgA AAC AAg ggT gTT TT-3'
